# Supplementary material for: In vitro immune-enhancing effects of Platycodon grandiflorum combined with Salvia plebeian via MAPK and NF-κB signaling in RAW264.7 cells
Source: PLoS One. 2024 Feb 2;19(2):e0297512. doi: 10.1371/journal.pone.0297512 (PMC10836713; doi:10.1371/journal.pone.0297512)

S1. Supplementary Materials-Original western blot gel image data

In vitro immune-enhancing effects of *Platycodon grandiflorum* combined with *Salvia plebeian* via MAPK and NF-κB signaling in RAW264.7 cells

Replication 1

- **p-NF-κB-p65**

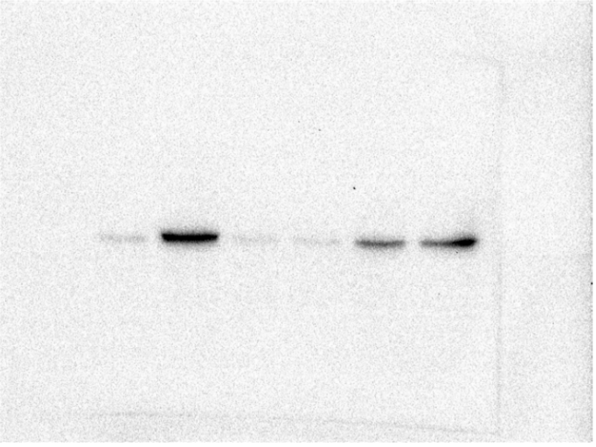

- **p-p38**

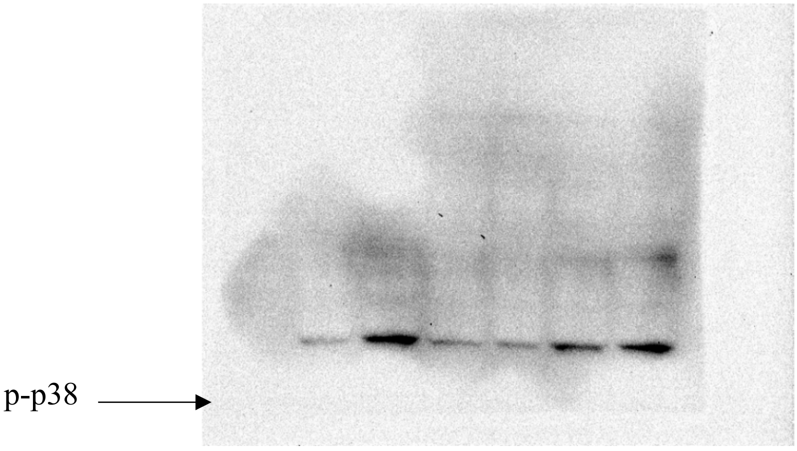

p-p38 →

- **p-JNK**

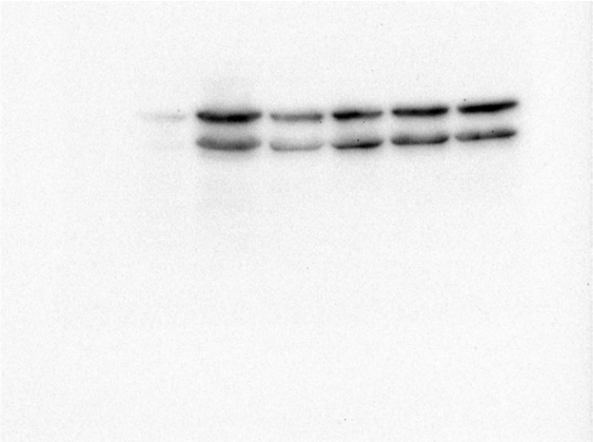

- **p-ERK 1/2**

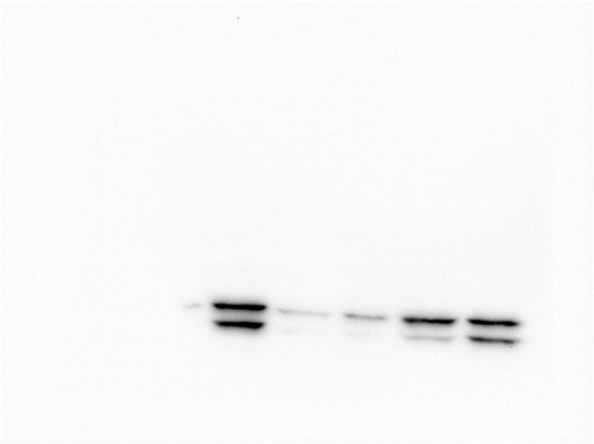

- **COX-1**

COX-1
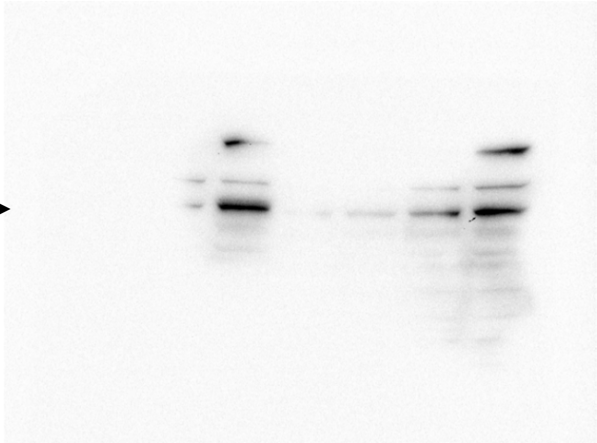

- **COX-2**

COX-2
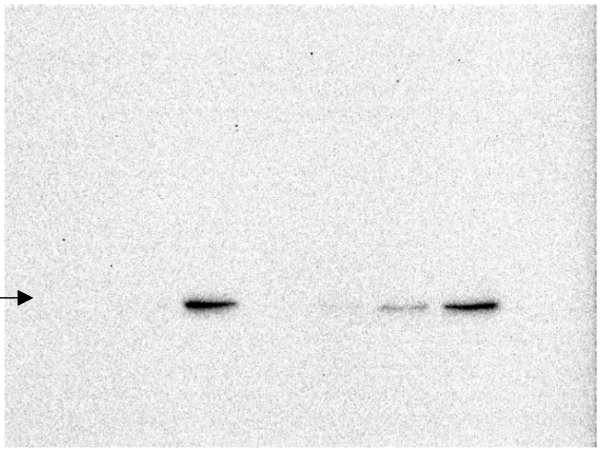

- **iNOS**

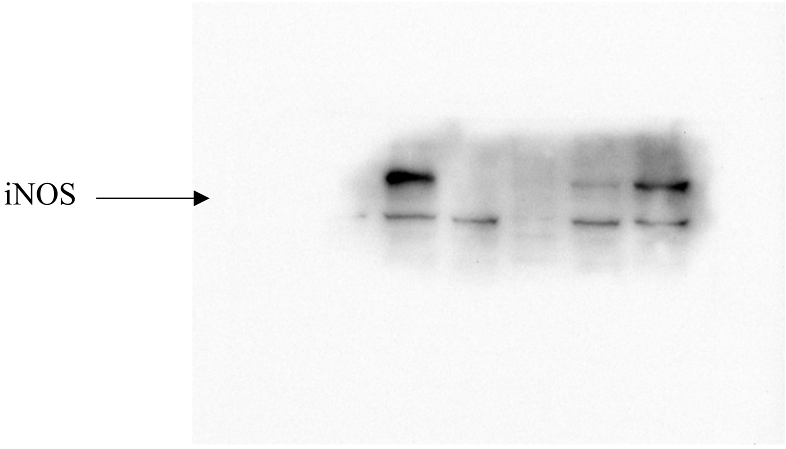

- **α-tubulin**

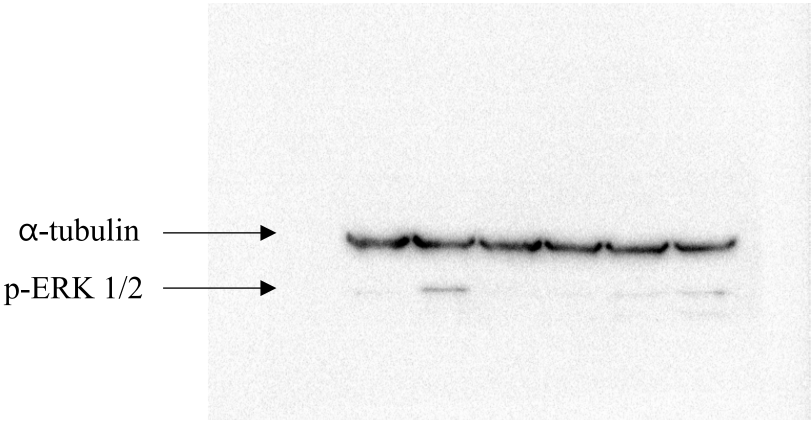

<u>Replication 2</u>

- **p-NF-κB-p65**

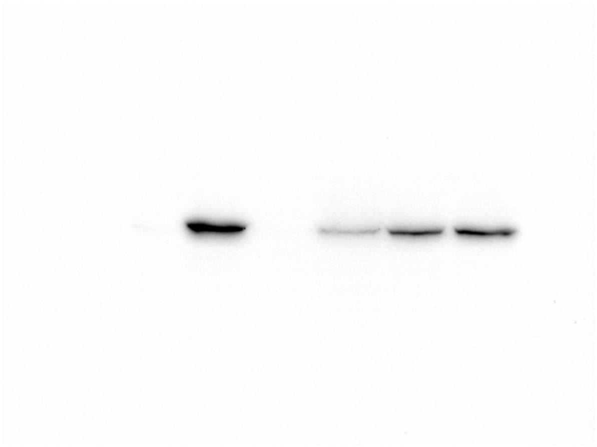

- **p-p38**

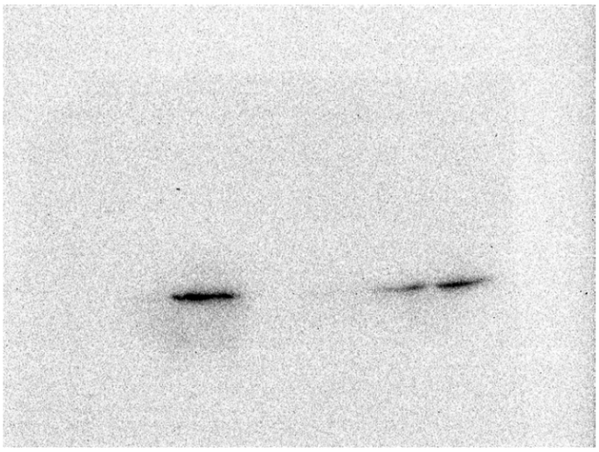

- **p-JNK**

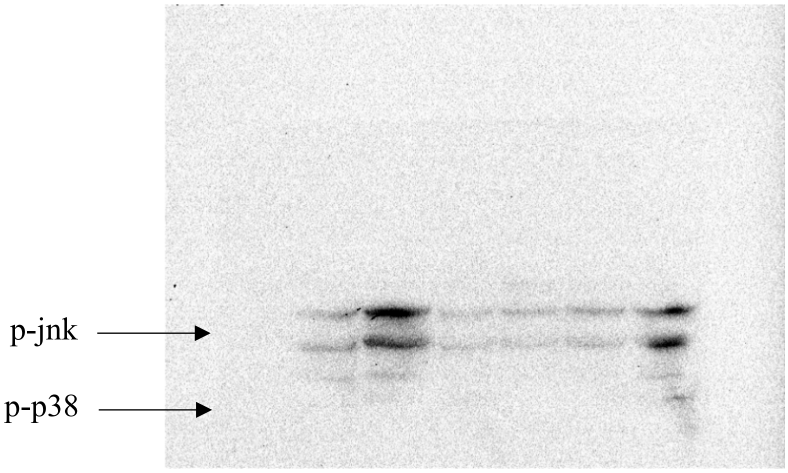

- **p-ERK 1/2**

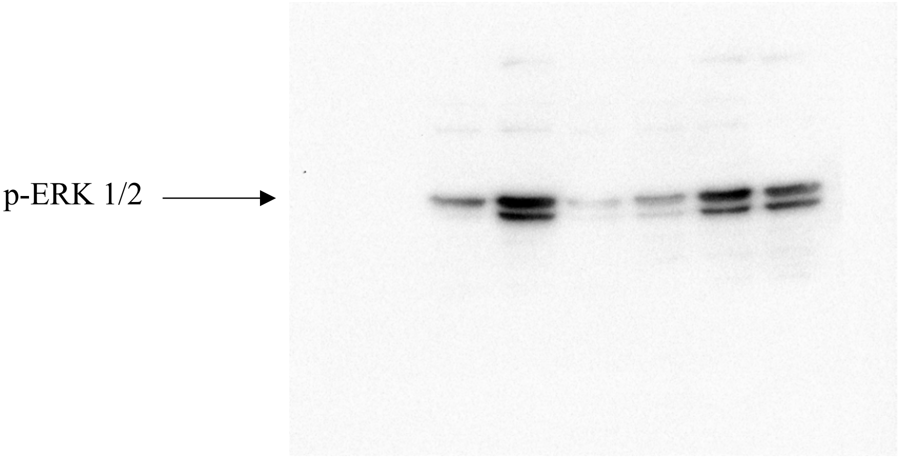

- **COX-1**

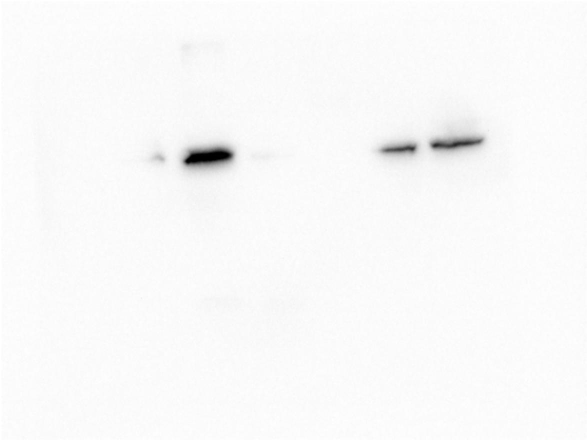

- **COX-2**

COX-2 →

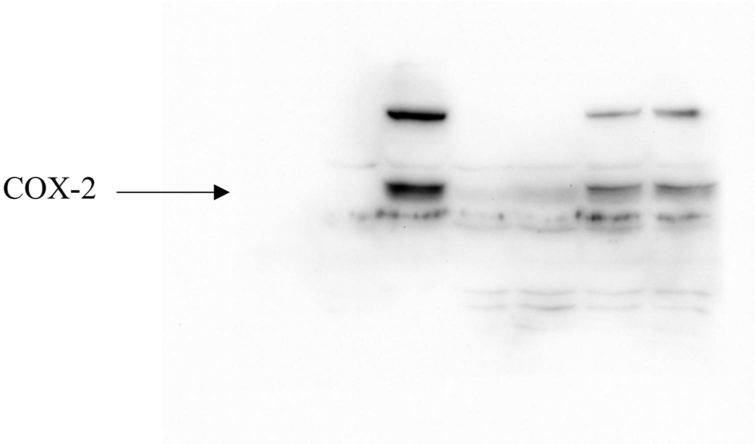

- **iNOS**

iNOS →

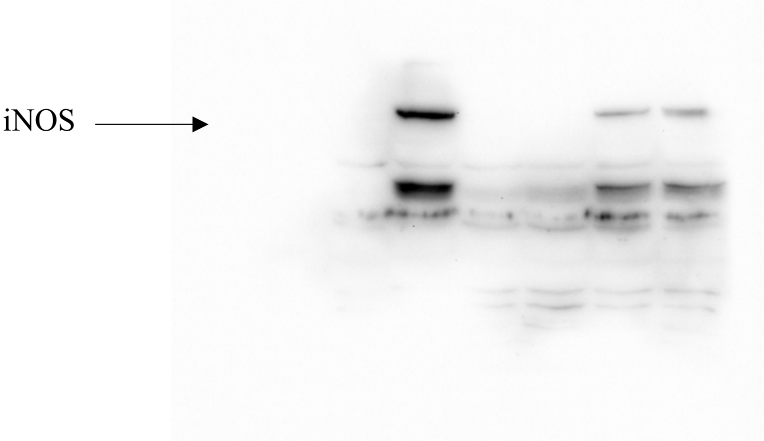

- **α-tubulin**

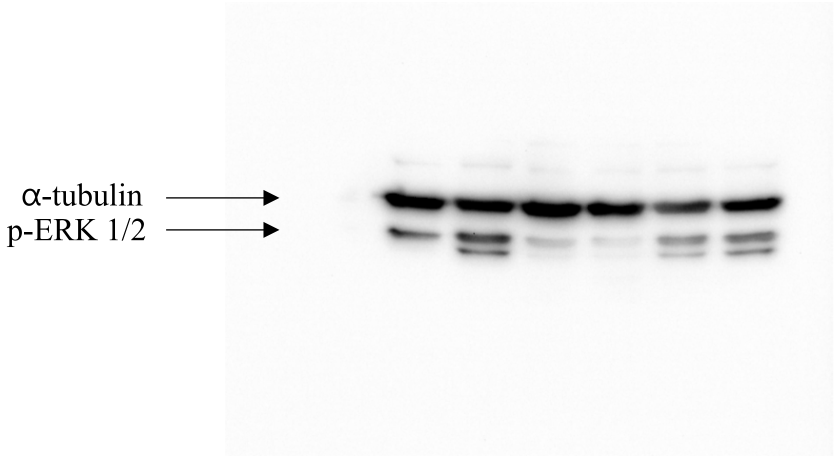

<u>Replication 3</u>

- **p-NF-κB-p65**

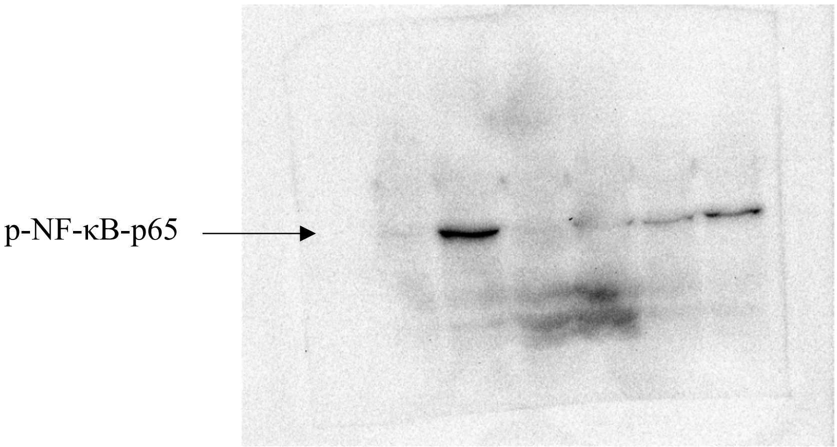

- **p-p38**

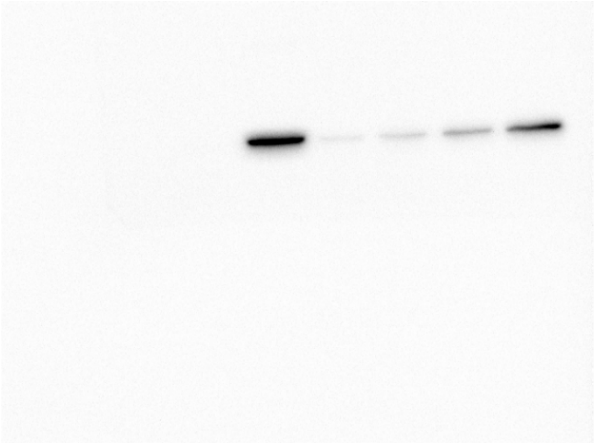

- **p-JNK**

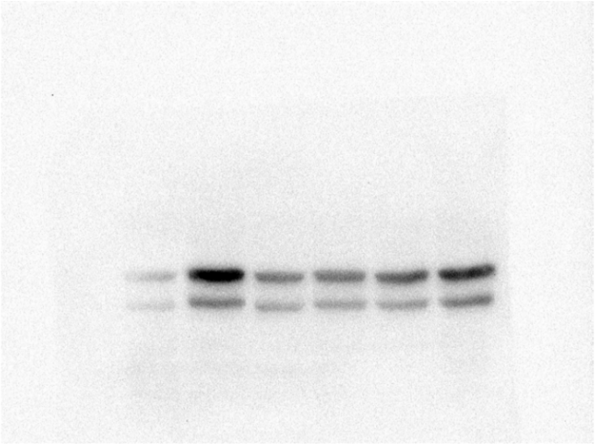

- **p-ERK 1/2**

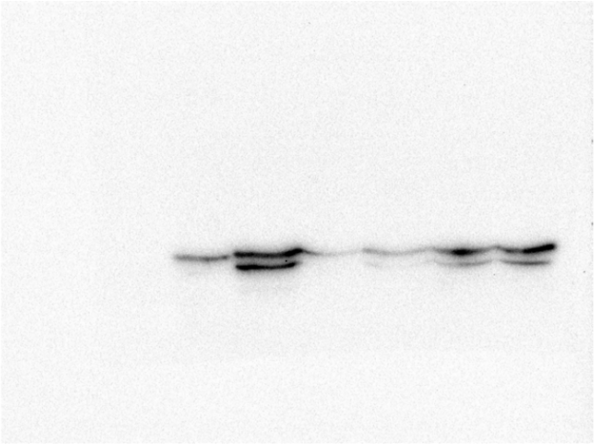

- **COX-1**

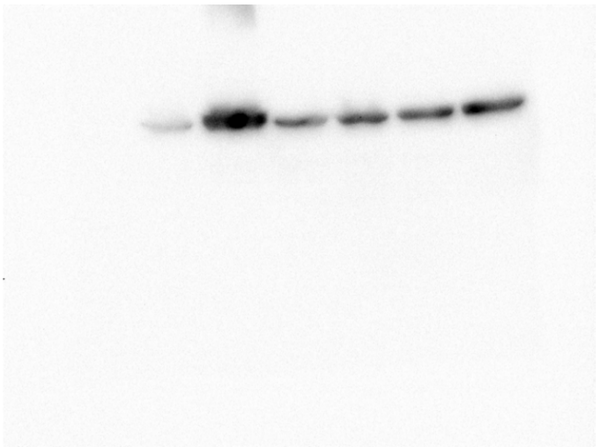

- **COX-2**

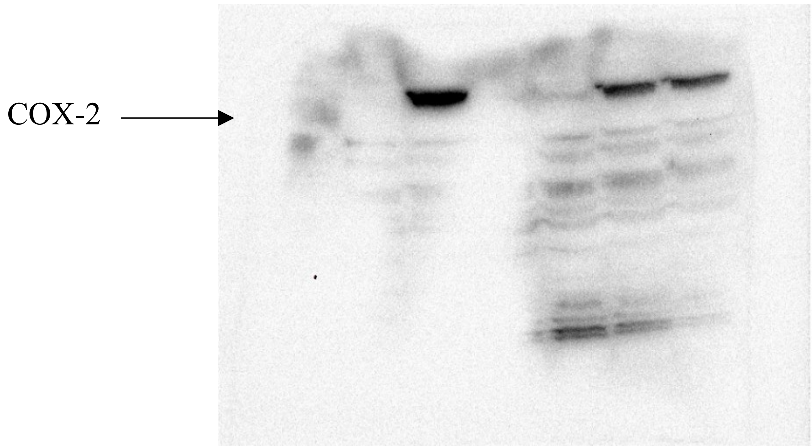

- **iNOS**

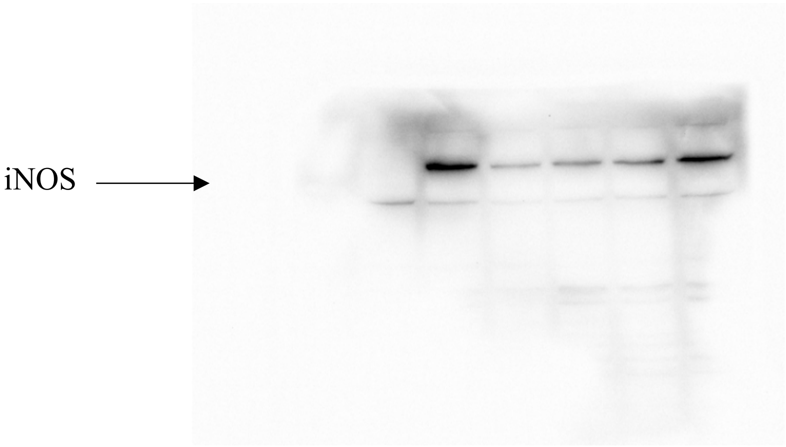

- **α-tubulin**

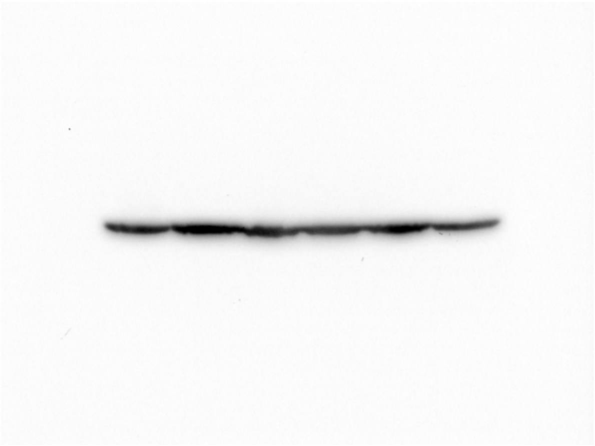

Supplement: S1 Fig — (PDF) [file pone.0297512.s001.pdf]
