# Supplementary material for: In vitro immune-enhancing effects of Platycodon grandiflorum combined with Salvia plebeian via MAPK and NF-κB signaling in RAW264.7 cells
Source: PLoS One. 2024 Feb 2;19(2):e0297512. doi: 10.1371/journal.pone.0297512 (PMC10836713; doi:10.1371/journal.pone.0297512)
Supplement: S1 File — (PDF) [file pone.0297512.s002.pdf]

S2. Supplementary Materials and methods

In vitro immune-enhancing effects of *Platycodon grandiflorum* combined with *Salvia plebeian* via MAPK and NF-κB signaling in RAW264.7 cells

Ultra-performance liquid chromatography–tandem mass spectrometry (UPLC–MS/MS) analysis of *P. grandiflorum* combined with *S. plebeian* (PSGP)

1. Sample preparation

40 mg of the freeze-dried PSGP was dissolved in 18% acetonitrile and filtered through a PVDF syringe filter (Whatman, USA) with a pore size of 0.45 μm. The sample (5 μL) was injected into UPLC–MS/MS systems.

2. Standards and stock solutions

Individual stock solutions of platycodin D (CAS: 58479-68-8) and platycoside E (CAS: 237068-41-6) were prepared at a concentration of 1,000 μg/mL by dissolving in 18% acetonitrile. The working solution was diluted with 18% acetonitrile to 0.78, 1.56, 3.13, 6.25, 12.5, 25, and 50 μg/mL, and a calibration curve was created using the working solution.

3. Chromatographic conditions

The samples were separated on a Capcell Pak C18 UG 120 column (5.0 μm, 4.6 × 250 mm). The condition of UPLC-MS/MS analysis was shown in Table S1. The platycodin D and platycoside E in PSGP were detected by comparing retention times of the standards, and their content was calculated using calibration curves based on linear correlations between concentrations of standards and peak area. The mass spectral parameters of the four coccidiostats are shown in Table S2.

Table S1. UPLC-MS/MS condition for analysis of Platycodin D and Platycoside E

**Parameter**		**Condition**		
UPLC	Column	Capcell Pak C18 UG 120, (5.0 m, 4.6 x 250 mm)		
	Column Temp.	30 °C		
	Mobile Phase (Gradient)	Time (min)	A[1)] (%)	B[2)] (%)
		0	82	18
		15	75	25
		30	70	30
		35	82	18
		40	82	18
	Flow rate	1.0 mL/min		
	Injection volume	5 µL		
MS/MS	Mode	ESI negative	Curtain gas	25 psi
	Gas 1	40 psi	Ion spray voltage	-4.5kV
	Gas 2	60 psi	CAD	6 eV

[1)] 0.1% formic acid in DW

[2)] Acetonitrile

Table S2. The parameters of mass spectrometer for analyzing Platycodin D and Platycoside E

**Compound**	**Q1**	**Q3**	**DP**	**EP**	**CE**	**CXP**
Platycodin D1	1223.3	469.2	-126	-10.0	-81.0	-10.0
Platycodin D2	1223.3	681.5	-120	-10.0	-90.0	-10.0
Platycoside E1	1547.7	469.1	-149	-11.0	-95.0	-16.0
Platycoside E2	1547.7	1005.6	-194	-11.0	-93.0	-44.0

Note: First quadrupole (Q1), third quadrupole (Q3), de-clustering potential (DP), entrance potential (EP), collision energy (CE) and collision cell exit potential (CXP)
