# Supplementary material for: In vitro immune-enhancing effects of Platycodon grandiflorum combined with Salvia plebeian via MAPK and NF-κB signaling in RAW264.7 cells
Source: PLoS One. 2024 Feb 2;19(2):e0297512. doi: 10.1371/journal.pone.0297512 (PMC10836713; doi:10.1371/journal.pone.0297512)
Supplement: Attachment — Submitted filename: Response to Reviewer 2 Comment_WJP.docx [file pone.0297512.s003.docx]

**Response to Reviewer 2 Comments**

Reviewer #2: The authors investigated the immune enhancing effects of PGSP via MAPK and NF-KB signaling in RAW264.7 cells. They related the genetic expression (mRNA) and protein levels of inflammatory markers (COX-2, TNF-alpha, IL-1B, IL-6, iNOS, etc) as basis for immune enhancing effects. They also attempted to describe the mechanisms of stimulation as being through phosphorylation of ERK, JNK, p38 and NF-kBp65. However, there are major concerns to the method use in this study.

1. The LPS control is at a concentration of 1ug/ml while the PGSP extracts are at 250-1000ug/ml. The results show that at 250ug, the levels of inflammatory markers (iNOS and COx2) on WB is almost nil (see figure 4c). PGSP extracts do not seem to stimulate inflammatory markers at low doses. Comparing their data with a control at a low dose is not a very good basis for comparison.

- LPS has been used as a positive control to stimulate the macrophage cells, and this concentration has been used for immune response research.
- Many previous researches like the followings have shown to use this concentration even though the concentration between the control group and samples is different.

**References**

- Zhou, Y., Qian, C., Yang, D., Tang, C., Xu, X., Liu, E. H., ... & Zhao, Z. (2021). Purification, structural characterization and immunomodulatory effects of polysaccharides from *Amomum villosum* Lour. on RAW 264.7 macrophages. Molecules, 26(9), 2672.
- Lin, S., Li, H. Y., Yuan, Q., Nie, X. R., Zhou, J., Wei, S. Y., ... & Wu, D. T. (2020). Structural characterization, antioxidant activity, and immunomodulatory activity of non-starch polysaccharides from *Chuanminshen violaceum* collected from different regions. International Journal of Biological Macromolecules, 143, 902-912.
- Tran, T. H. M., Mi, X. J., Huh, J. E., Mitra, P. A., & Kim, Y. J. (2023). *Cirsium japonicum* var. *maackii* fermented with *Pediococcus pentosaceus* induces immunostimulatory activity in RAW 264.7 cells, splenocytes and CTX-immunosuppressed mice. Journal of Functional Foods, 102, 105449.
- Jiang, S., Yin, H., Qi, X., Song, W., Shi, W., Mou, J., & Yang, J. (2021). Immunomodulatory effects of fucosylated chondroitin sulfate from *Stichopus chloronotus* on RAW 264.7 cells. Carbohydrate Polymers, 251, 117088.
- In addition, the results were corrected by comparing the significant differences within all treatments even though the signal is not strong and other previous reports have shown like our data.

**References**

- Lee, J., Kim, S., & Kang, C. H. (2022). Immunostimulatory activity of lactic acid bacteria cell-free supernatants through the activation of NF-κB and MAPK signaling pathways in RAW 264.7 cells. Microorganisms, 10(11), 2247.
- Jung, J. I., Lee, H. S., Kim, S. M., Kim, S., Lim, J., Woo, M., & Kim, E. J. (2022). Immunostimulatory activity of hydrolyzed and fermented *Platycodon grandiflorum* extract occurs via the MAPK and NF-κB signaling pathway in RAW 264.7 cells. Nutrition Research and Practice, 16(6), 685-699.
- Kim, J. S., Lee, E. B., Choi, J. H., Jung, J., Jeong, U. Y., Bae, U. J., ... & Lee, S. H. (2023). Antioxidant and immune stimulating effects of *Allium cepa* skin in the raw 264.7 cells and in the C57BL/6 mouse immunosuppressed by cyclophosphamide. Antioxidants, 12(4), 892.
- Gao, X., Qi, J., Ho, C. T., Li, B., Mu, J., Zhang, Y., ... & Xie, Y. (2020). Structural characterization and immunomodulatory activity of a water-soluble polysaccharide from *Ganoderma leucocontextum* fruiting bodies. Carbohydrate polymers, 249, 116874.
- Cho, H. Y., Lee, J. E., Lee, J. H., Ahn, D. U., & Paik, H. D. (2023). The immune-enhancing activity of egg white ovalbumin hydrolysate prepared with papain via MAPK signaling pathway in RAW 264.7 macrophages. Journal of Functional Foods, 103, 105487.
- Thanks.

The authors did not also show the levels of cytotoxicity due to this high dose extract except cell counts for cell viability. There are inflammatory markers of cell death and apoptosis (eg PD1). It would be good to show the levels of such markers in the study.

- Thank you very much for your valuable comment and suggestion.
- In this current study, the purpose of the experiment was focused on testing the percentage of cell viability and finding the concentration of the extract that does not cause cell death and is effective in increasing immunity.
- Additionally, previous many references such as the followings have shown the cytotoxicity similar to ours.

**References**

- Eo, H. J., Shin, H., Song, J. H., & Park, G. H. (2021). Immuno-enhancing effects of fruit of *Actinidia polygama* in macrophages. Food and Agricultural Immunology, 32(1), 754-765.
- Tran, T. H. M., Mi, X. J., Huh, J. E., Mitra, P. A., & Kim, Y. J. (2023). *Cirsium japonicum* var. *maackii* fermented with *Pediococcus pentosaceus* induces immunostimulatory activity in RAW 264.7 cells, splenocytes and CTX-immunosuppressed mice. Journal of Functional Foods, 102, 105449.
- Dong, Z., Zhang, M., Li, H., Zhan, Q., Lai, F., & Wu, H. (2020). Structural characterization and immunomodulatory activity of a novel polysaccharide from *Pueraria lobata* (Willd.) Ohwi root. International journal of biological macromolecules, 154, 1556-1564.
- He, P., Pan, L., Wu, H., Zhang, L., Zhang, Y., Zhang, Y., ... & Zhang, M. (2022). Isolation, identification, and immunomodulatory mechanism of peptides from *Lepidium meyenii* (Maca) protein hydrolysate. Journal of Agricultural and Food Chemistry, 70(14), 4328-4341.
- Park, C., HwangBo, H., Lee, H., Kim, G. Y., Cha, H. J., Choi, S. H., ... & Choi, Y. H. (2020). The immunostimulatory effect of indole-6-carboxaldehyde isolated from *Sargassum thunbergii* (Mertens) Kuntze in RAW 264.7 macrophages. Animal Cells and Systems, 24(4), 233-241.

2. The authors claim that there are synergistic effects due to the two combined extracts. However, they fail to show data for levels of inflammatory markers for each of the extracts.

- In our study, the effect of *Platycodon grandiflorum* alone on NO production at the concentrations of 250-1000 μg/ml for pre-experiments (data not shown in the manuscript) were measured.

- Compared with LPS (100%), the treatment of *P. grandiflorum* alone has slightly increased NO production to be 8.05-8.96% (A), while the mixture of *P. grandiflorum* and *S. plebeian* (PGSP) showed NO production to be 11.27-81.19% (B), that data showed in manuscript. Therefore, the results indicated that the PGSP were high efficiency more than *P. grandiflorum* alone.

- In addition, many previous studies reported that the immune-enhancement activity of only mixture of various plants have been shown and do not perform the experiments separately with individual substances.

- Thanks.

**References**

- Choi, M. (2022). Immunity-enhancing effect of extracts extracted from leaves of *Rubia hexaphylla*, *Cymbopogon citratus*, and *Dioscorea japonica* for sustainable healthy life. Sustainability 2022, 14, 2804.
- Han, N. R., Kim, K. C., Kim, J. S., Ko, S. G., Park, H. J., & Moon, P. D. (2022). The immune-enhancing effects of a mixture of *Astragalus membranaceus* (Fisch.) Bunge, *Angelica gigas* Nakai, and *Trichosanthes Kirilowii* (Maxim.) or its active constituent nodakenin. Journal of Ethnopharmacology, 285, 114893.
- Kim, M. C., Lee, G. H., Kim, S. J., Chung, W. S., Kim, S. S., Ko, S. G., & Um, J. Y. (2012). Immune-enhancing effect of Danggwibohyeoltang, an extract from *Astragali Radix* and *Angelicae gigantis* Radix, *in vitro* and *in vivo*. Immunopharmacology and Immunotoxicology, 34(1), 66-73.
- Park, Y. M., Lee, H. Y., Shin, D. Y., Kim, D. S., Yoo, J. J., Yang, H. J., ... & Bae, J. S. (2022). Immune-Enhancing Effects of Co-treatment With *Kalopanax pictus* Nakai Bark and *Nelumbo nucifera* Gaertner Leaf Extract in a Cyclophosphamide-Induced Immunosuppressed Rat Model. Frontiers in Nutrition, 9.
- Shin, H. J., Gwak, H. M., Lee, M. Y., Kyung, J. S., Jang, K. H., Han, C. K., ... & Kim, S. H. (2019). Enhancement of respiratory protective and therapeutic effect of *Salvia plebeia* R. Br. extracts in combination with Korean red ginseng. Korean Journal of Medicinal Crop Science, 27(3), 218-231.

**Minor Concerns**
1. Why was the extract diluted in 1% FBS and P/S and not in plain medium?

- The reaction of NO production after adding Griess reagent causes to form a pink-red azo dye, and the pink color is the result of nitic accumulation. It is not led by the color of the RPMI (normally red). Also, as you know, phenol red has own pink color. It can inhibit the identification of NO production in case phenol red is included.

- Therefore, the extract was diluted in RPMI-1640 medium without phenol red, 1% FBS, or P/S, which did not have color.

- Thanks.

2. The authors need to state the concentration of the cells used for RNA extraction, Western blot as well as Macrophage phagocytosis assay.

- It was corrected.

- Thanks.
3. The authors did not state clearly how the mRNA expression analysis was done. They seem to use Actin as control but they did not say how many biological and technical replicates were done and how they analysed for mRNA expression.

- It was corrected.

- Thanks.
